# Highly efficient method for gene delivery into mouse dorsal root ganglia neurons

**DOI:** 10.3389/fnmol.2015.00002

**Published:** 2015-02-02

**Authors:** Lingli Yu, Florie Reynaud, Julien Falk, Ambre Spencer, Yin-Di Ding, Véronique Baumlé, Ruisheng Lu, Valérie Castellani, Chonggang Yuan, Brian B. Rudkin

**Affiliations:** ^1^Differentiation and Cell Cycle Group, Laboratoire de Biologie Moléculaire de la Cellule, UMR 5239, Centre National de la Recherche Scientifique, Ecole normale Supérieure de Lyon, University of Lyon 1 Claude Bernard, University of LyonLyon, France; ^2^Laboratory of Molecular and Cellular Neurophysiology, East China Normal UniversityShanghai, China; ^3^Joint Laboratory of Neuropathogenesis, Key Laboratory of Brain Functional Genomics, Chinese Ministry of Education, East China Normal University, Centre National de la Recherche Scientifique, Ecole Normale Supérieure de LyonShanghai, China; ^4^Centre de Génétique et Physiologie Moléculaire et Cellulaire, UMR Centre National de la Recherche Scientifique 5534, University of Lyon 1 Claude Bernard, University of LyonVilleurbanne, France

**Keywords:** dorsal root ganglion (DRG) neuron, transfection, electroporation, nucleofection, gene expression, primary neurons, EGFP expression, Nerve growth factor (NGF)

## Abstract

The development of gene transfection technologies has greatly advanced our understanding of life sciences. While use of viral vectors has clear efficacy, it requires specific expertise and biological containment conditions. Electroporation has become an effective and commonly used method for introducing DNA into neurons and in intact brain tissue. The present study describes the use of the Neon® electroporation system to transfect genes into dorsal root ganglia neurons isolated from embryonic mouse Day 13.5–16. This cell type has been particularly recalcitrant and refractory to physical or chemical methods for introduction of DNA. By optimizing the culture condition and parameters including voltage and duration for this specific electroporation system, high efficiency (60–80%) and low toxicity (>60% survival) were achieved with robust differentiation in response to Nerve growth factor (NGF). Moreover, 3–50 times fewer cells are needed (6 × 10^4^) compared with other traditional electroporation methods. This approach underlines the efficacy of this type of electroporation, particularly when only limited amount of cells can be obtained, and is expected to greatly facilitate the study of gene function in dorsal root ganglia neuron cultures.

## Introduction

Culture of primary cells has been extensively used to study neuronal survival, signal transduction, development, and neurite outgrowth. Gene transfer, through both viral and non-viral methods, has become a powerful technique to assess the effects of expression of selected genes. Adenovirus, herpes-simplex virus (HSV), lentivirus, and adeno-associated virus (AAV) have been reported to deliver transgenes both *in vivo* and *in vitro* (Glatzel et al., 2000; Chattopadhyay et al., 2005; Towne et al., 2009; Yu et al., 2011). While effective, these approaches are time-consuming, labor-intensive and carry some potential biohazard risk. Non-viral methods, which mainly include microinjection of DNA, biolistic, sonoporation, lipid- or chemical-based transfer or electroporation, offer faster and safer means for gene delivery (Table 1).

**Table 1:** **Comparison of non-viral methods commonly used to transfect mammalian neurons**.

**Non-viral method**	**Model**		**Maximal efficiency**		**Cell survival**	**References**
	**Precursors**	**Post-mitotic cells**	**DNA plasmids**	**RNAi**		
Ca^2+^phosphate/DNA co-precipitation		erHCn	Typically between 1 and 5%; 12 and 27% under highly controlled conditions	Efficient %n.c.	%n.c.	Goetze et al., 2004
Lipofection		nmCGn	Effective (≥50% Knock-down)	%n.c.	%n.c.	Butcher et al., 2009
		erHCn/erCn	20–25% / 25–30%^a^	–	100%^b^	Ohki et al., 2001; Dalby et al., 2004
		erHCn	–	73%^c^	89–96%	Tonges et al., 2006
Biolistics			Typically around 2%, rarely reaches 10% in cultured neurons, up to 34% in slice culture	–	%n.c.	Karra and Dahm, 2010
		nr/arDRGn/ SCGn	5%	–	%n.c.	Dib-Hajj et al., 2009
Sonoporation		DRGn	31%		35%	Lin et al., 2010
Electroporation “Nucleofection”		nr/ar/amDRGn/SCGn	5–20%	Efficient	%n.c.	Dib-Hajj et al., 2009
		ecDRGn			37%	Martinez and Hollenbeck, 2003
	mNSC		88%	Efficient >50%	78%	Bertram et al., 2012^d^
	hNPCs		10–20%		44%	Dieterlen et al., 2009
		arDRGn/erDRGn/ecDRGn/SCGn/ erHCn	39–42%		%n.c.	Chadborn et al., 2006; Jones et al., 2006; MacGillavry et al., 2009; Ketschek and Gallo, 2010; McCall et al., 2012; Pristera et al., 2012; Kirton et al., 2013

erHCn, embryonic rat hippocampal neurons; hNPCs, human neural progenitor cells; NSC, neural stem cells; CGns, cerebellar granule neurons; SCGn, superior cervical ganglion neurons. amDRGn, adult Mouse DRGn; emDRGn, embryonic mouse DRGn; arDRGn, adult Rat DRGn; nrDRGn, neonatal rat DRGn; ecDRGn, embryonic chick DRGn; %n.c., percent not communicated.

a Lipofectamine 2000™.

b The authors state that 100% of the cells in transfected cultures are viable.

c Stearyl-R8.

d Nucleofector® 4D.

Electroporation, particularly because of its ease of use, combined with efficient and precise targeting in space and time, has become an effective method for introducing DNA into neurons in culture, slices and in intact neural tissue of Xenopus, chick and mouse (Kawabata et al., 2004; Falk et al., 2007; Saijilafu and Zhou, 2011). Dorsal root ganglia (DRG)-derived sensory neurons that are selectively sensitive to Nerve growth factor (NGF), Brain derived neurotrophic factor (BDNF) and Neurotrophin-3 (NT3), provide an excellent model in which to study the mechanisms of axonal regeneration, neurotrophin signaling, peripheral nervous system development and peripheral neuron disease (Melli and Hoke, 2009; Newbern et al., 2011). Existing electroporation methods of dissociated DRG neurons require relatively large amounts of cells. As example, the Amaxa Nucleofector system, one of the best known and commonly performed transfection methods now in the labs, requires 1 × 10^6^ DRG cells in 100 μl for each electroporation [e.g., Chick DRG (Chadborn et al., 2006); Manufacturer's instructions]. This is a major obstacle when working with embryos. Additionally, the low survival and transfection rate with the standard Amaxa system are of major concern. Though encouraging results have been reported recently with adult Rat DRG using the 4D-Nucleofector system-X from Lonza (McCall et al., 2012), electroporation of dissociated DRG neurons, from young mouse embryos, whose cell number is limiting, remains a challenge.

Herein, we describe an optimized procedure for isolation and dissociation of mouse embryonic DRG neurons and their electroporation with plasmid DNA (≈5 kb and ≈4.7 kb) in a rapid and highly effective manner with efficiencies comparable to those reported for viral infection, while maintaining high viability and transgene expression.

## Materials and methods

### Reagents

Poly-L-Lysine (P1399, Sigma-Aldrich), Laminin (L2020, Sigma-Aldrich), calcium/magnesium free Hank's Balanced Salt Solution (HBSS) (14170, Life technologies), Trypsin (T5266, Sigma-Aldrich, pH = 7.2), DNAse 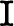 (DN25, Sigma-Aldrich), Nutrient Mixture F-12 (21765, Life technologies), Fetal calf serum (FCS) (10270106, Life technologies), penicillin-streptomycin (P/S) (15140, Life technologies), Nerve growth factor (NGF) (mouse 2.5S; N-100, Alomone Labs), Cytosine-arabinoside “Ara-C” (C1768, Sigma-Aldrich), anti-β III tubulin antibody (SC-53140, Santa Cruz), Donkey anti-mouse antibodies conjugated to Alexa Fluor® 488 (715-545-151, Jackson ImmunoResearch), Vectashield (Vector, H-1000).

### Animals

Time pregnant OF1 mice were purchased from Charles River (France). The mice were euthanized by cervical dislocation according to ethical committee recommendations (Authorization # 007050).

### Plasmids

pEGFP-N1 (4.7 kb) was from Lonza; pCDNA3.1-Cav-1 fused to RFP (pCDNA3.1-Cav-1-RFP (5.25 kb), Addgene Plasmid 14434) was a gift from R. E Pagano; (Sharma et al., 2004); pCDNA3.1-RFP was modified by removing the Cav1 coding sequence from pCDNA3.1-Cav-1-RFP. An Endofree DNA purification kit (Nucleobond XtraMAxi, ref. 740424.10) was used to isolate GFP, RFP and CAV1-RFP plasmids according to manufacturer's protocol. Plasmids were resuspended in culture grade PBS (Ca^++^, Mg^++^-free; 14190-094, Life technologies) at 3–5 μg/μl.

### Preparation of coated coverslips

Coverslips were soaked in 96% ethanol for 30 min, then washed with distilled water and/or air dried. Poly-L-lysine (50 μg/ml) was spread equally over the surface of the coverslip, followed by incubation for 3 h at room temperature or overnight at 4°C. They were subsequently rinsed with distilled water, then 50 μl Laminin (10 μg/μl) in HBSS was applied on a coverslip. A second coverslip was placed, coated side down, on the first one, then allowed to sit overnight at 37°C. After two washes with HBSS, the coverslips were placed one per well into a 24-well tissue culture plate. Wells were then filled with 500 μl of culture medium without antibiotics and plates pre-incubated in a humidified 37°C/5% CO_2_ incubator prior to use.

### Isolation and dissociation of dorsal root ganglia from mouse embryos

We used a modified protocol, adapted from a previously described procedure for isolation and culture of cortical neurons (Castellani et al., 2004; Falk et al., 2014). Time pregnant mice at E14.5 were sacrificed by cervical dislocation and embryos were removed from the uterus kept on ice. Heads were removed from the embryos in ice cold HBSS (Ca^++^/Mg^++^-free) and the bodies washed in the same. Both Male and Female embryos were used. The embryos were pinned down on the silicon-coated dish with dorsal side up in cold HBSS with 2% glucose. The skin of the embryo was cut along the dorsal midline. The cartilage was then cut along the midline and the spinal cord removed with the forceps tip by sliding them along the spinal cord. The meninge was removed to visualize the DRG along the vertebral canal, which were taken out one by one using forceps. Buffer in the dissection dish was changed for every embryo. Typically up to 18 DRG are isolated per embryo.

Dissected DRG from three E14.5 embryos were transferred into an eppendorf tube containing 270 μl of ice cold in Ca^++^/Mg^++^-free HBSS to which 30 μl of trypsin (25 mg/ml) were added to yield a final concentration of ≈2.5 mg/ml (Total volume ≈300 μl) and incubated for 10 min at 37°C. 10 μl of DNAse 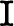 (0.1 mg/ml) were added to a final concentration of 0.033 mg/ml, and returned to 37°C for an additional 10 min incubation, gently shaking the tube approximately every 3 min to equally distribute the DRG. Neutralization of the trypsin was performed by adding 30 μl of FBS followed by 500 μl of culture medium (F12, 10% FBS without antibiotics). DRG explants were centrifuged 3 min at 900 rpm (fixed angle rotor, radius 60 mm, Eppendorf minispin) and Trypsin-containing medium was removed. Mechanical dissociation of cells was carried out using a fire polished glass Pasteur pipette with a bulb, by triturating approximately 15 times in 200 μl F12, 10%FCS without antibiotics. Cells were counted to evaluate the number of conditions possible (6–9 × 10^4^ cells per condition) before re-suspension in electroporation buffer. An average of 27 × 10^4^ cells were isolated per embryo (range 18–37 × 10^4^).

In order to achieve a high survival rate of the cells, it is essential to keep the dissection within 1 h after sacrifice of the mother.

### Electroporation

After counting the cells, 1.3 ml Ca^++^/Mg^++^-free HBSS was added and the cells centrifuged for 2 min at 300 rpm (fixed angle rotor, radius 60 mm, Eppendorf minispin). The cells were resuspended with half of the electroporation buffer R (Invitrogen) that would be needed for the number of conditions estimated. Since cells are lost in this process, they were counted again to adjust the amount to 6–9 × 10^4^ cells per condition with electroporation buffer R (10 μl total per electroporation). 10 μl of cells were gently pipetted into each microfuge tube, then mixed with the appropriate plasmids, i.e., pEGFP (Lonza) (0.5 μg) and pCDNA3.1-Cav-1 fused to RFP (pCDNA3.1-Cav-1-RFP) or pCDNA3.1-RFP (1 or 2 μg). Cells were immediately electroporated using the Neon® transfection system. Electrical parameters, i.e., voltage and pulse length, were varied in order to find the optimal conditions. Cells should not be kept in the electroporation buffer for longer than 10 min. After electroporation, cells were immediately transferred into two wells with coated coverslips, containing medium without antibiotics, pre-warmed to 37°C. The culture dishes were gently rocked to ensure even distribution of the cells, which were then left to settle for 1 h in the CO_2_ incubator at 37°C. NGF (50 ng/ml final) and antibiotics (1% final) were then added to the medium and 12 h later, Ara-C (10 μM final) was added to remove contaminating glial cells.

### Imaging and data analysis

Control and caveolin-1-RFP-expressing cultures from the same dissociation batch were fixed in 2% paraformaldehyde for 14 -16 h at 4°C, 24 and 48 h after addition of NGF, and transgene expression was analyzed. Phase contrast and fluoresence images were acquired using a 20x objective as a 3 × 3 montage. 4 montages of 9 images were collected in different regions of the coverslips to minimize bias due to different cell density (Zeiss AxioImager, Z1 upright). The number of transfected neurons per image was calculated using the Cell Counter plugin for ImageJ.

For determination of the impact of electroporation on cell survival and differentiation, non-transfected control and 1 μg RFP/0.5 μg GFP expressing cultures from the same dissociation batch were fixed in 2% paraformaldehyde for 14–16 h at 4°C, 24 and 48 h after addition of NGF. Phase contrast images were acquired using a Zeiss AxioImager, Z1 upright, 20x objective. Approximately 14 images were collected in different regions of the coverslips to minimize bias due to different cell density. The number of surviving neurons (round, bright clear) per image was calculated using the Cell Counter plugin for ImageJ. The overall neurite outgrowth per image was calculated using the NeuronJ plugin for ImageJ (1 pixel = 0.318 μm).

For immunolabeling of neurons, cells were fixed in 2% paraformaldehyde for 14–16 h at 4°C and permeabilized at room temperature (RT) for 5 min with 0.1% Triton X-100 in PBS. After washing with PBS at RT, cells were blocked for 1 h at RT with 10% normal goat serum/1% BSA/0.1% Triton in PBS. Samples were incubated with anti-β III tubulin mouse monoclonal antibody (Santa Cruz, SC-53140) 14–16 h at 4°C and visualized with secondary antibodies conjugated to Alexa 488 (Goat-anti-mouse, Jackson ImmunoResearch, 115-485-003) to label neurons. Coverslips were mounted in Vectashield. Images were acquired using Olympus FluoView FV10i. Data acquisition was performed with Olympus FluoViewsoftware.

### Statistics

Statistical significance between various groups was tested using the Mann-Whitney test.

## Results

### Optimization of transfection conditions for dissociated DRG neurons

To determine the protocol that would produce the highest possible transfection efficiency and greatest survival, the DRG neurons were electroporated with 1 μg pEGFP, varying voltage, duration and number of pulses. The three pulse parameters listed in Table 2 were initially tested in this work since they have been successfully applied to hippocampal, cortical and neuronal stem cells from rat embryos (Manufacturer's protocol). After 24 h, cell viability and transfection efficiency were assessed by visual inspection. As shown in Table 2, electroporation with two pulses at 1300 V with a pulse width of 20 ms, produced high transfection efficiencies and cell viability. Therefore, this setting was utilized for subsequent experiments.

**Table 2:** **Optimization of electroporation**.

**Conditions**	**Survival**	**Efficiency**
1. 1500 V 20 ms 1 pulse	++	+
2. 1600 V 10 ms 3 pulses	+	++
3. 1300 V 20 ms 2 pulses	++	++

DRG neurons (6–9 × *10^4^* cells for each condition) were electroporated with pEGFP (1 μg, Lonza). Electroporation conditions were optimized for voltage, duration and number of pulses. After 24 h, cell viability and transfection efficiency were assessed by visual inspection.

The amount of pCDNA3.1-Cav-1-RFP or pCDNA3.1-RFP DNA was then varied between 1 and 2 μg for which the volume was normalized to 10% of the total reaction volume in distilled water; 0.5 μg pEGFP was co-transfected for neurite tracing in transfected neurons. 24 and 48 h post-transfection, the cells were fixed and processed for microscopic analysis. As shown in Table 3, over 50% of the neurons were positive for GFP, while increasing RFP or Cav1-RFP plasmid concentration did not result in significantly higher transfection efficiency. On average, all conditions considered, a transfection efficiency of 54 ± 1.1% (Average ± S.E.M.) was achieved at 24 h. After 48 h, 67 ± 1.4% of fluorescent neurons were observed, indicating the survival of transfected neurons.

**Table 3:** **Percentage of RFP and Cav1-RFP positive cells with different expression constructs**.

**Time point**	**Construct**	**Percentage of GFP(+) neurons/Total neurons (%)**	**Percentage of RFP(+) neurons/total neurons (%)**	**Percentage of Cav1-RFP(+) neurons/Total neurons (%)**
24 h	0.5 μg GFP/1 μg RFP	58.1 ± 3.7	57.9 ± 3.6	
	0.5 μg GFP/1 μg Cav1-RFP	51.5 ± 2.0		53.9 ± 1.7
	0.5 μg GFP/2 μg RFP	55.0 ± 3.1	55.2 ± 3.1	
	0.5 μg GFP/2 μg Cav1-RFP	50.5 ± 3.1		52.6 ± 3.6
48 h	0.5 μg GFP/1 μg RFP	62.6 ± 2.5	62.6 ± 2.5	
	0.5 μg GFP/1 μg Cav1-RFP	67.0 ± 3.7		80.3 ± 2.8
	0.5 μg GFP/2 μg RFP	68.6 ± 3.3	68.0 ± 3.1	
	0.5 μg GFP/2 μg Cav1-RFP	60.1 ± 5.2		64.5 ± 4.8

Cells were transfected with pEGFP and each expression construct for RFP at two different concentrations and the number of transfected cells was determined by visual inspection. Results were obtained from three independent experiments. Data represent Mean ± S.E.M.

**Figure 1:**
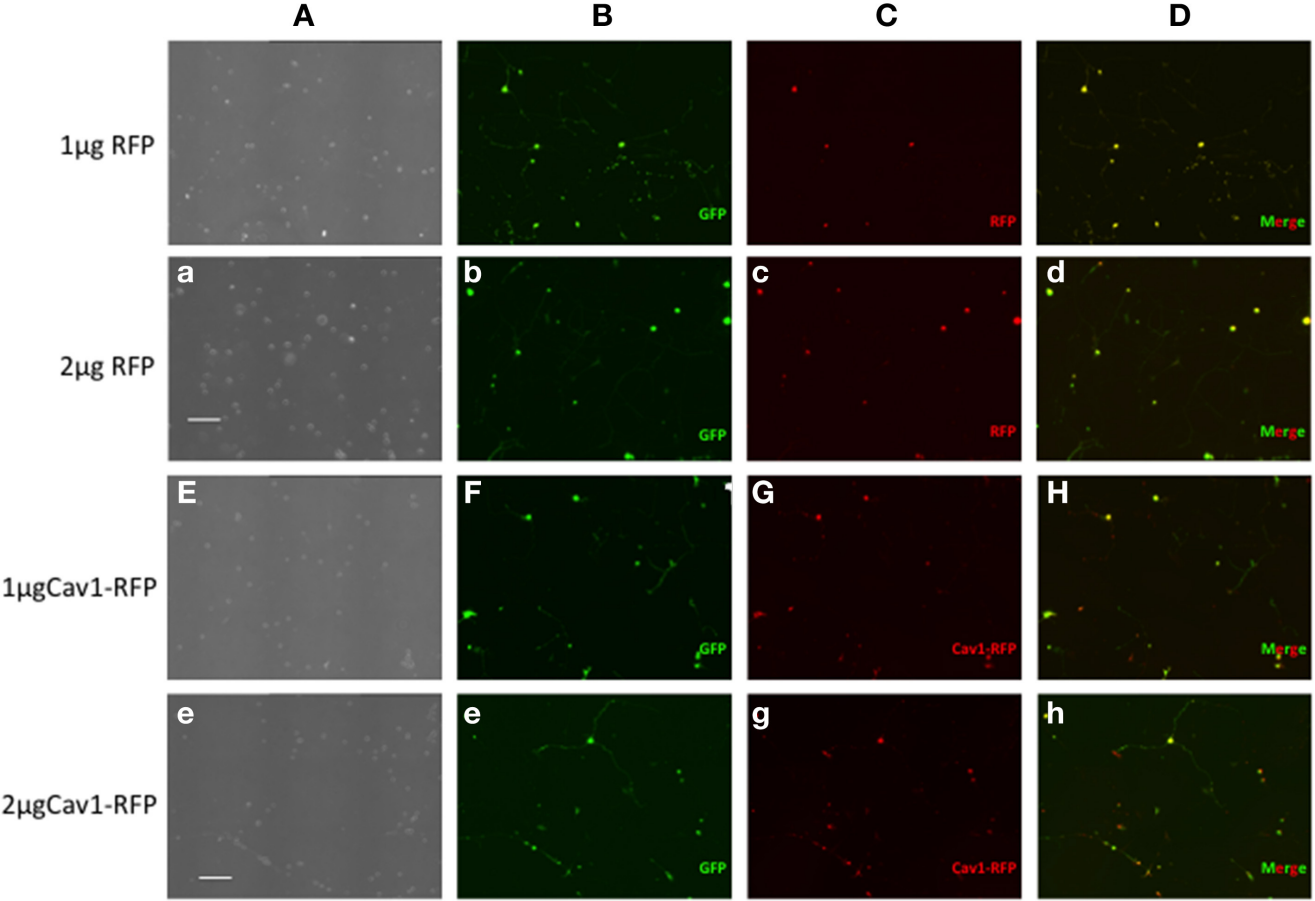
**Expression of fluorescent proteins in DRG neurons**. Neon transfection leads to efficient electroporation of E14.5 DRG neurons. Neurons were transfected with 0.5 μg EGFP along with 1 or 2 μg of RFP or 1 or 2 μg Cav1-RFP. 24 h after electroporation, cells were fixed and observed under the microscope. Column A: Phase contrast image. Column B: GFP fluorescence. Column C: RFP fluorescence. Column D: Merge of GFP and RFP Fluorescence. Scale Bars represent 200 μm.

**Figure 2:**
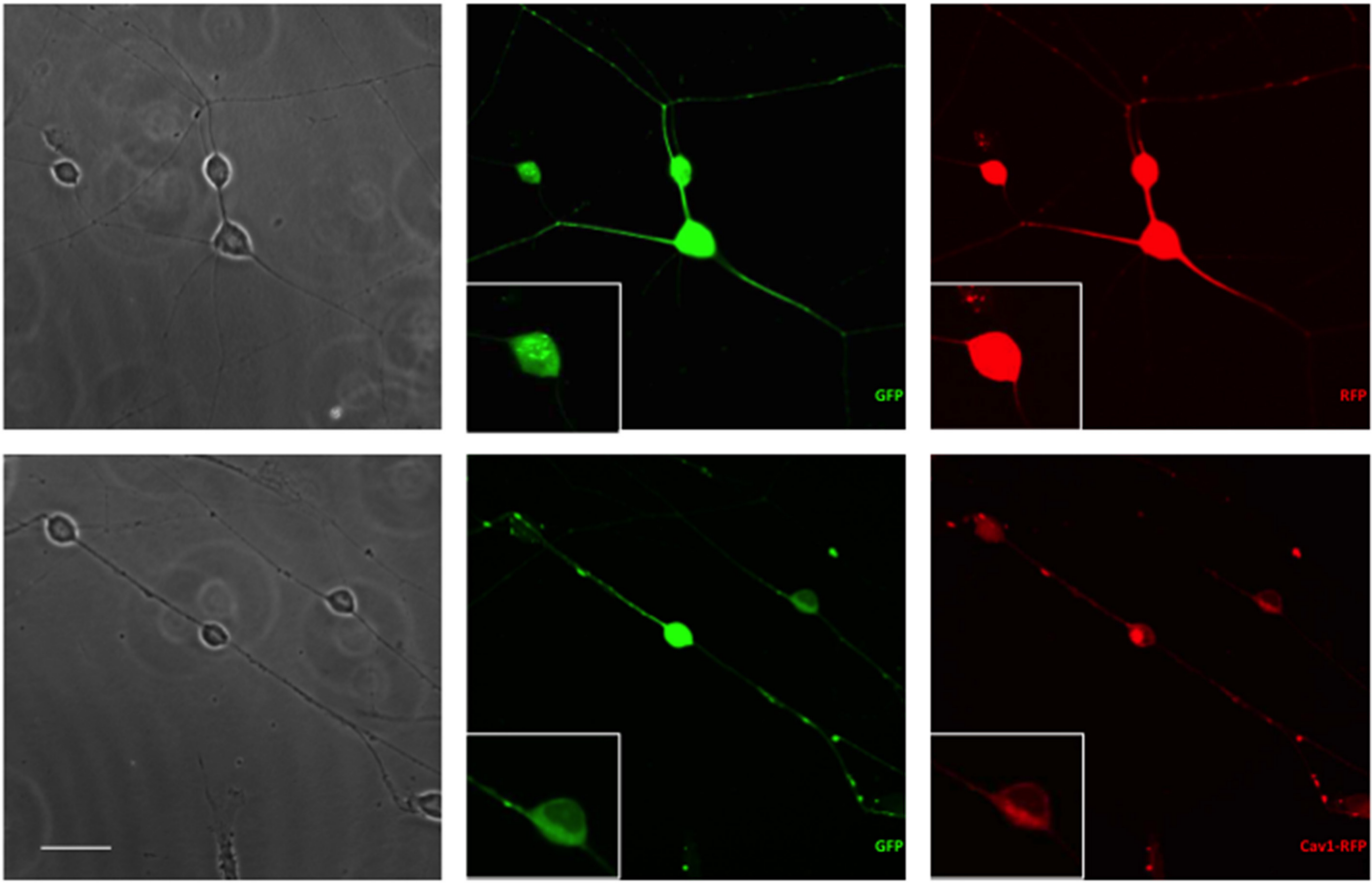
**Expression of fluorescent proteins in DRG neurons**. Neurons were transfected with 1 μg RFP or Cav1-RFP combined with 0.5 μg EGFP. 24 h after electroporation, cells were fixed and observed under the microscope. Scale Bar represents 50 μm.

Essentially all of the cells carrying GFP were also transfected with RFP or Cav1-RFP (Figure 1). Both EGFP and RFP were visualized over the entire length of the axons, whereas Cav1-RPF was predominantly expressed in the cell body (Figure 2).

Other parameters, such as the cell density and plasmid purity are also important issues. Use of low cell numbers 2 × 10^4^ to 4 × 10^4^ cells/electroporation, resulted in decreased transfection efficiency (Not shown). Also, avoiding multiple centrifugations during the procedure effectively reduces cell loss while enhancing viability. The use of endo-free plasmids is highly recommended as they result in high cell viability and thereby complement the transfection efficiency.

Finally, as mentioned in the Materials and Methods section, it is particularly important for the high transfection efficiency and survival to use DRG isolated within 1 h after sacrifice of the mother.

### The survival and differentiation of DRG neurons after electroporation

**Figure 3:**
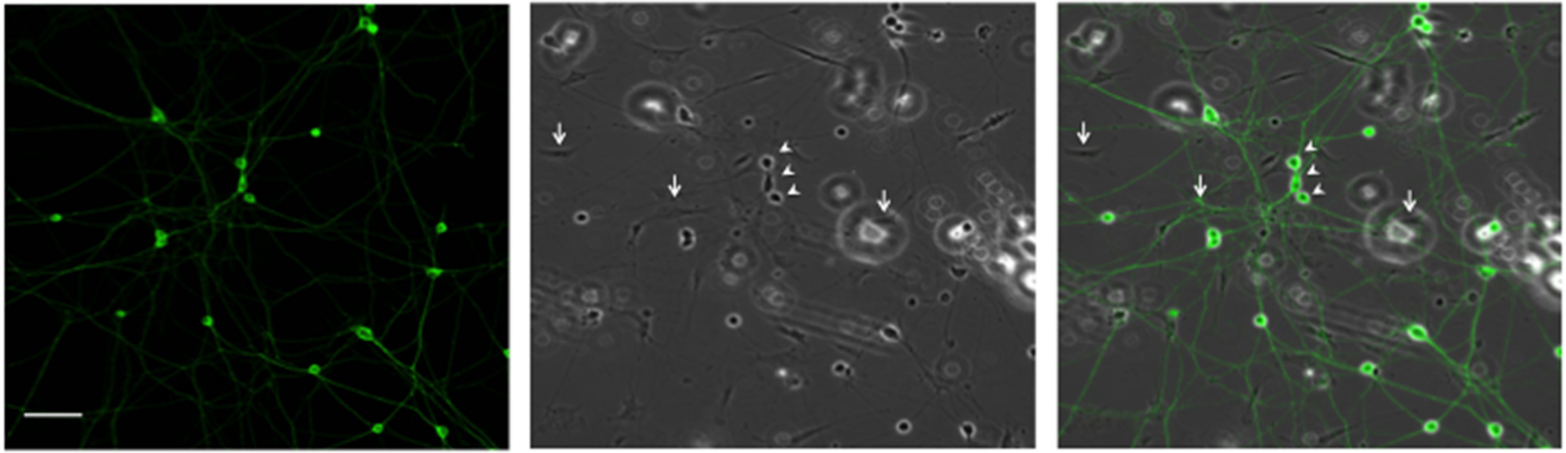
**Representative DRG neurons in culture**. DRG neurons were dissected and maintained in culture. 24 h later, cells were fixed and labeled with anti-β III tubulin antibody. Arrows and arrowheads point, respectively, to glial and neuronal cells. Scale Bar represents 100 μm.

**Figure 4:**
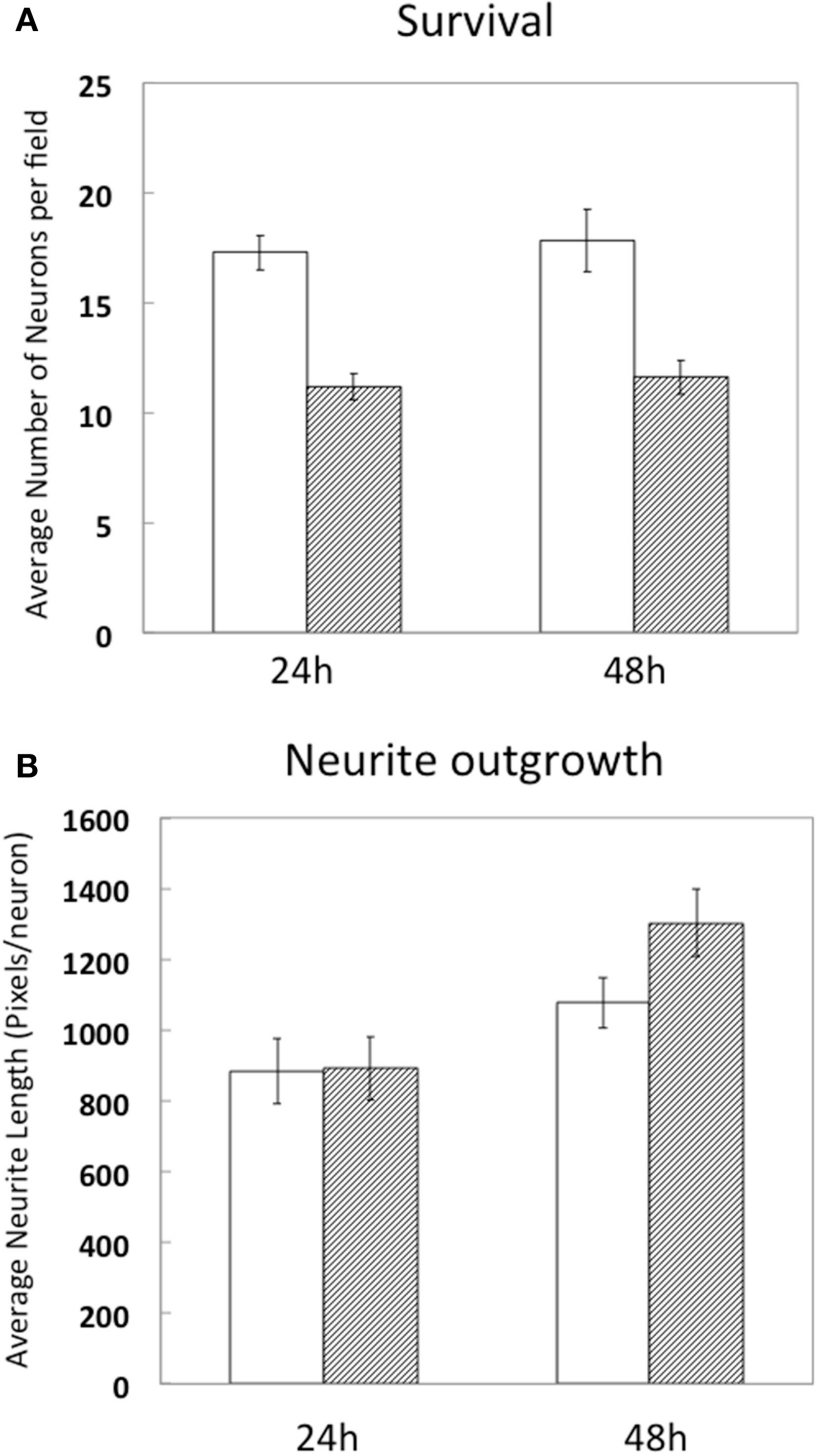
**Survival and differentiation of DRG neurons after electroporation**. DRG neurons were prepared and directly cultured without transfection (white bars) or transfected with 1 μg RFP combined with 0.5 μg EGFP (hashed bars). 24 and 48 h after electroporation, cells were fixed and neuron survival **(A)** and average neurite length per neuron (1pixel = 0.318 μm) **(B)** were calculated compared to naïve, non-transfected neurons. Results were obtained from three independent experiments. Data represent mean ± S.E.M. derived from 40 images representative of 692 non-transfected neurons (24 h) and 39 images representative of 439 GFP/RFP expressing neurons and 29 images representative of 523 non-transfected neurons and 31 images representative of 360 GFP/RFP expressing neurons of naïve, non-transfected controls (48 h).

Since high electroporation efficiencies are often achieved at the expense of cell survival, we evaluated survival subsequent to Neon® electroporation. Under phase-contrast, healthy neuronal cells are easily distinguished since their cell bodies are round and phase bright and display extensive neurite outgrowth, as shown in Figure 3. As shown in Figure 4A, cultures of transfected neurons had approximately 35% fewer neurons than cultures of non-transfected neurons. Between 24 and 48 h, neuron number was maintained, under both conditions, indicating that the transfected neurons survived as well as non-transfected. 24 and 48 h post-electroporation, neuronal survival rates were 65% of naïve, non-transfected controls.

The extent of average neurite growth was as robust in transfected cultures as with non-transfected, increasing in both between 24 and 48 h (Figure 4B). The average neurite length in non-transfected cultures at 24 h was 885 ± 93 pixels per neuron vs. 892 ± 90 for transfected. At 48 h, average neurite length was 1077 ± 71 pixels/neuron in non-transfected and 1302 ± 95 in transfected cultures.

## Discussion

Electroporation, because of its ease of use, reproducibility and relatively high efficiency, has been developed and widely used for introducing various molecules into cells. In this study, we describe an optimized method for gene delivery into embryonic DRG neurons, with high transfection efficiency of >60%, and low cytotoxicity as reflected in a 2/3 survival rate and robust differentiation, comparable to non-transfected cultures.

Primary neurons present a particular challenge to successful gene transfer. Adenovirus (Ad), adenovirus-associated virus (AAV) was successfully used for gene transfer *via in vivo* injection of mice (Glatzel et al., 2000). Herpes simplex virus (HSV) (Storey et al., 2002), and lentivirus (Yu et al., 2011) have been applied in rat DRG cells. Although they effectively deliver genes into DRG cells, several viruses can affect sensory neuron physiology (Fukuda and Kurata, 1981; Maehlen et al., 1991; Farkas et al., 1994) and thus limit their use in some experiments.

The Neon® transfection system applied to embryonic DRG neurons as described herein, resulted in a mean transfection efficiency for DNA plasmids well over 60% in DRG neurons, along with high viability and robust differentiation comparable to non-transfected cultures at 48 h which, to our knowledge, is the highest reported for a non-viral transfection method, yielding levels comparable to viral infection (Table 4).

**Table 4:** **Electroporation of DRG**.

**Parameter**	**Martinez**	**Ketschek**	**Chadborn**	**Dib-Hajj**	**Kirton**	**Lonza**	**MacGillavry**	**McCall**	**Pristera**	**YU**
Year	2003	2010	2006	2009	2013	–	2009	2012	2012	This work
Species	Chick	Chick	Rat	Rat or Mouse	Rat	Mouse	Rat	Rat	Rat	Mouse
Sex M: Male; F: Female	–	–	–	n.c.	M	–	M	n.c.	n.c.	M/F
Age	embryo E9-E12	embryo E7	embryo E18	neonatal or adult	7 day old	embryonic or adult	adult	adult	adult	embryo E14.5
Cell number	2.5–12.5 × 10^6^	2 × 10^6^ ^c^	1 × 10^6^	2 × 10^6^	2 × 10^6^	5 × 10^4^ or 2 × 10^4^	0.2–1 × 10^6^ ^c^	3 × 10^5^	5 × 10^4^	6–9 × 10^4^
DNA (μg)	0.5–1.25	10^d^	5	2+10^a^; 1+5^b^	2	0.1–0.6	n.c.^e^	2	≥1	1.5–2.5
Electroporation volume (μl)	25	100^c^	100	100	100	20	20^c^	20	10	10
Apparatus	BTX Electro Square Porator T820 Genetronics	Nucleofector® II Lonza	Nucleofector®	Nucleofector®	Nucleofector® 2B	Nucleofector® II - “S”	Nucleofector® 96-well system	Nucleofector®-4D X-unit	Neon® Life Technologies	Neon®
Program	Phosphate Buffered Saline 1mM CaCl2 5.5 mM glucose	G-013	G-13	G-013 rat DRG O-003 mouse DRG	O-03	SNC Basic Neuro Program 6 Nucleofector Solution	c	DR114/P3 Buffer	Buffer R	Buffer R
Voltage V/cm	1120	–	–	–	–	–	–	–	1200	1300
Pulse duration (ms)	5	–	–	–	–	–	–	–	20	20
Number of Pulses	1	–	–	–	–	–	–	–	2	2
Efficiency (48 h)	12%	n.c.	n.c.	5–20%	n.c.	≈60%^f^	siRNA ≥50%	40%	n.c.	67%
Survival	n.c.	n.c.	n.c	n.c.	n.c.	≈40%^f^	n.c.	18–27%	n.c.	>60%

Summary of existing methodology reports describing electroporation/nucleofection of DRG from embryonic Chick, Rat (embryonic and adult) and Mouse (adult), compared to the study presented herein. Unless otherwise stipulated, efficiency and survival are at 48 h post-electroporation; n.c., not communicated.

a adult Rat or mouse (10 = Carrier DNA);

b neonate Rat (5 = Carrier DNA);

c authors communicated electroporation was performed according to manufacturer's protocol;

d manufacturer suggests 1–2 μg DNA;

e expression plasmids and siRNA were used. No indication of amount of DNA;

f 24 h post-electroporation.

Usually, 1–2 × 10^6^ cells are required for each electroporation to achieve reasonable number of transfectants and good cell survival in the first and second generation electroporation apparatuses. This is due to the necessity to have an optimal cell density and the original large volume (100 μl) of the electroporation chambers. The advent of smaller chamber volumes allows a significant reduction in cell number. As few as 3 × 10^5^ cells have been successfully used with the Nucleofector 4D in a volume of 20 μl/1 mm chamber with an efficiency of 40%, which is comparable to the results obtained with the 100 μl chamber (Manufacturer's protocol) and survival rates of up to almost 30% (McCall et al., 2012). The Neon® system was reported to have been successfully used DRG neurons isolated from adult Rats, with a cell number (5 × 10^4^) and program similar to the one we have found to be optimal for DRG's isolated from embryonic mice. There is no data however on the actual efficiency or survival. Using the approach presented herein 6–9 × 10^4^ cells were sufficient for each transfection of DRG neurons from mouse embryos. Thus, with the cells obtained, for example, from one E14.5 embryo, four electroporations can be performed, largely expanding the experimental conditions, while reducing the total number of embryos needed for certain types of experiments. The other main advantage of our method is that, when electroporated with mixed plasmids comprising EGFP and RFP at a ratio of 1:2–1:4, we found that essentially all of the neurons expressed both of these fluorescent proteins. As EGFP was sufficiently detectable the entire length of the axons, one can easily analyze the effect of transfected genes with a GFP-based imaging system. By combining other plasmid constructs, such as TET-ON/OFF system (Shaikh and Nicholson, 2006) and using cell type-specific promoters (Hitoshi et al., 1999; Boulaire et al., 2009; Gulick and Robbins, 2009), it should be possible to express transgenes in a spatiotemporal manner. In summary, the transfection protocol presented herein for embryonic DRG neurons, employed the Neon® transfection system and effectively enabled heterologous gene expression in DRG neurons.

While viral transduction/infection has yielded significant advances, it remains more labor-intensive than electroporation of purified plasmids. Furthermore, there are safety issues regarding the types of genes that can be introduced under standard laboratory conditions, e.g., oncogenes or genes that inhibit apoptosis or modulate autophagy. Clearly, implementation of the protocol proposed herein alleviates both issues, enabling investigation of the impact of such genes or modulators thereof on DRG survival, differentiation and function.

## Author contributions

Lingli Yu, Florie Reynaud, Julien Falk, Chonggang Yuan, and Brian B. Rudkin—participated in the conception, execution and analysis of experiments and in the writing of the manuscript. Ambre Spencer, Yin-Di Ding, Véronique Baumlé, and Ruisheng Lu participated in the conception, execution and analysis of initial experiments. Valérie Castellani oversaw the experiments at CGphiMC, participated in the data analysis, revision and final approval of the text. All authors agree to be accountable for all aspects of the work.

### Conflict of interest statement

The authors declare that the research was conducted in the absence of any commercial or financial relationships that could be construed as a potential conflict of interest.
