# Erratum on: Highly efficient method for gene delivery into mouse dorsal root ganglia neurons

**DOI:** 10.3389/fnmol.2015.00016

**Published:** 2015-05-18

**Authors:** Frontiers Production Office

**Affiliations:** Frontiers Production OfficeFrontiers, Lausanne, Switzerland

**Keywords:** dorsal root ganglion (DRG) neuron, transfection, electroporation, nucleofection, gene expression, primary neurons, EGFP expression, nerve growth factor (NGF)

Reason for Erratum:

Due to a typesetting error, there was an error in the order of affiliations for authors Chonggang Yuan and Brian B. Rudkin. The affiliations now appearing as Chonggang Yuan^1,3*^ and Brian B. Rudkin^2,3*^, should be: Chonggang Yuan^2,3*^ and Brian B. Rudkin^1,3*^

1. Differentiation and Cell Cycle Group, Laboratoire de Biologie Moléculaire de la Cellule, UMR 5239, Centre National de la Recherche Scientifique, Ecole normale Supérieure de Lyon, University of Lyon 1 Claude Bernard, University of Lyon, Lyon, France
2. Laboratory of Molecular and Cellular Neurophysiology, East China Normal University, Shanghai, China
3. Joint Laboratory of Neuropathogenesis, Key Laboratory of Brain Functional Genomics, Chinese Ministry of Education, East China Normal University, Centre National de la Recherche Scientifique, Ecole Normale Supérieure de Lyon, Shanghai, China

This error does not change the scientific conclusions of the article in any way. The publisher apologizes for this mistake.

